# Psychometric Properties of the Sexually Aggressive Behaviors Scale: Factor Structure, Reliability, and Construct Validity in a Sample of Portuguese Female College Students

**DOI:** 10.1177/0306624X221113535

**Published:** 2022-07-21

**Authors:** Pedro J. Rosa, Nélio Brazão, Joana Carvalho

**Affiliations:** 1 Lusófona University, HEI-Lab, Lisbon, Portugal; 2 Instituto Superior Manuel Teixeira Gomes (ISMAT), Portimão, Portugal; 3 University of Coimbra, Portugal; 4 Portuguese Ministry of Justice, Lisbon, Portugal; 5 Porto University, Portugal

**Keywords:** construct validity, factor structure, female college students, psychometrics, reliability, sexual violence

## Abstract

This study investigates the psychometric properties of the Sexually Aggressive Behaviors Scale (SABS) in a sample of 1,632 heterosexual Portuguese female college students, aged between 18 and 39 years old (*M* = 22.40; *SD* = 4.31). The internal structure of the scale was investigated, along with analyses of the internal consistency, and validity evidence in relation to external variables. Participants completed an online survey that was disseminated using Portuguese universities’ mailing lists and social networks. Results supported a single factor solution consisting of 10 items. Evidence was gathered in favor of the internal consistency and convergent/discriminant validity in relation to other variables, such as rape myths, psychopathic traits, and perception of intimacy. This study adds to the evidence of the SABS being an appropriate evaluation tool with female college students, allowing the rigorous assessment of sexual violence as committed by community women against a reluctant male partner.

## Introduction

Sexual violence (SV) among young adults and college students is a worldwide public health problem (Kaya et al., 2020). Although men are more sexually aggressive than women, recent studies have shown that women also resort to aggressive means to initiate sexual intercourse with an unwilling male partner, namely sexual coercion (Bouffard et al., 2016; Marshall et al., 2021; Miller et al., 2017).

SV perpetrated by women against men is believed to be non-trivial in prevalence among college students. Nevertheless, we cannot directly compare data from the different studies, as researchers use distinct questions to capture SV (Carvalho & Brazão, 2021). Women report using distinct “hands-off” strategies, including psychological manipulation, verbal pressure, blackmailing, taking advantage when the man is intoxicated by alcohol or other drugs or using a position of power and authority (Carvalho et al., 2018; Carvalho & Nobre, 2016; Turchik, 2012). However, “hands-on” strategies, that include threatening and/or using physical force, have also been reported (Carvalho & Nobre, 2016; Carvalho et al., 2018). Prevalence rates of male college students reporting women’s SV have varied from 5% to 38.5% (Badenoch, 2015; Hines et al., 2012). While the lowest rates correspond to behaviors representing severe forms of SV (hands-on strategies), the highest rates represent the less severe forms, that is, the hands-off strategies. Further, studies conducted with heterosexual couples showed that between 2.8% and 37% of college men reported being forced into sex by women (Aosved et al., 2011; Artime et al., 2014; Lehrer et al., 2015).

As for women, 26% reported using sexual coercion (Struckman-Johnson et al., 2003) and 5% endorsed using physical force to attain sex (i.e., rape; Monson et al., 2009). In a review conducted by Williams et al. (2008), the prevalence rates of SV by female college students against men ranged from 2.1% to 46.2%. More recently, findings from two independent samples revealed that 32.7% to 35.8% of female college students used sexually aggressive behaviors to initiate sexual intercourse with an unwilling man/partner. These included verbal tactics (46.2%–72.3%), use of power/authority (34.1%–46.5%), and physical force (13.1%–19.8%; Carvalho & Nobre, 2016; Carvalho et al., 2018). Taken together, these findings suggest that women and men presented a similar pattern of sexual initiation by coercive means, with both using similar levels of physical force and power/authority, while men reported to use significantly more verbal tactics (Carvalho & Brazão, 2021).

In the past few years, only a few studies have targeted the putative markers behind SV perpetrated by women. Some of these studies characterized the cultural/social factors underlying women’s SV, whereas others focused on their psychological profile (individual and interpersonal factors). At a cultural/social level, the endorsement of rape myths has been identified as a motivational factor for SV. In accordance with Burt (1980), rape myths are “prejudicial, stereotyped, or false beliefs about rape, rape victims, and rapists” (p. 217), which may result in a rape-supportive climate hostile for victims. Therefore, rape myths may reinforce what is and is not SV, as well as who is a “credible” victim, which has implications for victims, offenders, and society (Walfield, 2021). From a cognitive perspective, these rape myths could be conceptualized as cognitive distortions that female offenders use when processing information, to justify their criminal conduct and/or to minimize the consequences of their own behavior. In fact, it is recognized from the literature on female criminal samples, that the endorsement of a distorted cognitive style about the sexual offences and the victims is a motivational factor for SV (Beech et al., 2009; Carvalho et al., 2018). Nevertheless, most research about rape myths has been conducted with a focus on male offenders (Edwards et al., 2011), and studies on the acceptance of these rape myths in community samples of sexually aggressive women has yet to be conducted.

At an individual and interpersonal level, psychopathic traits and intimacy difficulties have been identified as dynamic risk factors for women’s SV, respectively (Gannon & Cortoni, 2010; Gannon et al., 2008). The link between psychopathy and SV is well established, both in criminal samples of male and female offenders (Bouffard et al., 2016; Gannon & Cortoni, 2010; Miller et al., 2017). Concerning intimacy, findings with female sexual offenders showed that difficulties in establishing intimate/romantic relationships may be a crucial motivational factor for SV. In this sense, it has been argued that female sex offenders may use sex to regulate emotional states or fulfil intimacy needs (e.g., Gannon et al., 2008; Nathan & Ward, 2002). Despite the available findings, the link between intimacy deficits and women’s SV needs further investigation, and studies testing the association between psychopathy, intimacy deficits, and SV in women from community samples are, to our best knowledge, lacking.

Concerning methods aimed at assessing SV perpetration, most instruments may be gendered-biased, considering their emphasis on male perpetration and female victimization (e.g., Budd, 2017). To our best knowledge, there is only one measure specifically designed to assess sexually aggressive behaviors as committed by community women against a reluctant man: The Sexually Aggressive Behaviors Scale (SABS; Anderson, 1996). The SABS was designed to assess sexual aggression as initiating sexual contact (kissing, fondling, and intercourse) by using sexual coercion (e.g., threatening to end a relationship, verbal pressure, or lying), sexual abuse (e.g., sex with a minor by an adult at least 5 years older that the minor, by inducing intoxication, or by using a position of power or authority), or psychically forced sex (i.e., by the threat of psychical force, actual physical force, or the use of a weapon). The instrument was adapted from the 13-item Sexual Experience Survey (Koss & Oros, 1982), and adaptations included a change in gender specificity (from man as initiator to woman as initiator), from respondent as receiver of initiation to respondent as initiator, the inclusion of behaviors other than sexual intercourse (e.g., kissing and fondling), and additional questions regarding sexual behaviors (e.g., while someone was intoxicated) and motivations (e.g., to retaliate or to hurt someone else). The SABS is composed of 26 items, and each question asks respondents how many times they had ever initiated sexual contact by engaging in each behavior (i.e., sexual coercion, sexual abuse, and physical force; Anderson, 1996). Through an exploratory factor analysis, Anderson (1996) found this measure to be unifactorial (sexual aggression), with acceptable internal consistency. Nevertheless, to our best knowledge, no further study tested the psychometric properties of the SABS. Considering that this is the only measure specially designed to assess the sexually aggressive behaviors perpetrated by community women (see Measures section), more studies are needed to establish SABS as a robust instrument assessing SV perpetrated by women. Considering the prevalence of SV perpetrated by young women and college students, further studies with the SABS are of the utmost importance.

This research proposed to evaluate the psychometric properties of the SABS, using both exploratory and confirmatory factor analyses and exploring the one-factor solution found by Anderson (1996). In particular, the internal structure and consistency of the SABS was investigated. The validity evidence on relation to external variables, namely rape myths, psychopathic traits, and perception of intimacy, was also explored.

We expected that the one-factor structure would present a good fit to the data. Concerning construct validity and considering the available literature and/or results from previous studies (e.g., Beech et al., 2009; Bouffard et al., 2016; Gannon & Cortoni, 2010; Gannon et al., 2008), the SABS scores were expected to associate positively with rape myths and psychopathic traits, and negatively with the perception of intimacy (personal validation, communication, and openness to outward).

## Materials and Methods

### Participants and Procedures

Participants in this study included 1,632 heterosexual Portuguese female college students, aged between 18 and 39 years old (*M* = 22.40; *SD* = 4.31). Most participants were attending undergraduate courses (*n* = 1,572; 96.3%), were single (*n* = 1,472; 90.2%), and had one current sexual partner (*n* = 1,148; 70.3%). On average, participants had the first sexual intercourse experience at 17 years old (*M* = 17.29; *SD* = 2.10).

This study is part of a larger research project on female sexual offending behavior and was approved by the Ethics Committee of the School of Psychology and Life Sciences, Lusófona University. Participants completed an online survey (data were stored in a secure server, handle only by the authors). The research project was publicly advertised as a study on female sexuality (rather than a study on women’s sexual violence). The study was disseminated using Portuguese universities’ mailing lists and social networks, between February and December 2019. All participants reported informed consent and did not receive any incentives for participating in the study.

### Measures

*Sexually Aggressive Behaviors Scale* (SABS; Anderson, 1996). The SABS is a 26-item self-report measure specifically developed for assessing sexually aggressive behaviors perpetrated by community women (i.e., from the general population) against men, thus capturing the specific dynamics underlying female sexual offending behavior. This measure is composed of 12 critical items (assessing sexually aggressive behaviors) and 14 filing items (not theoretically relevant, used to *hide* the critical items). The critical items are aimed to assess the frequency of the following attempted sexually aggressive behaviors: sexual coercion (i.e., attempting sexual interaction by means of verbal pressure, blackmailing, or using psychological manipulation; e.g., “How many times have you attempted to have sexual contact with a man by pressuring him with verbal arguments?”); sexual abuse (i.e., using a position of power or authority to attempt sexual contact; e.g., “How many times have you attempted to have sexual contact with a man by getting him drunk or high?”); and physical force (i.e., threatening or effectively using physical force to attempt sexual intercourse; e.g., “How many times have you attempted to have sexual contact with a man by using some degree of physical force?”). Respondents are asked to identify the frequency of each behavior, using a dichotomous scale (0 = the behavior has never occurred or 1 = the behavior has occurred at least once). The original version of the scale revealed an acceptable internal consistency, with an alpha of .75 (Anderson, 1996).

The SABS was translated and adapted into Portuguese following a translation and back-translation procedure (Hambleton et al., 2005). The translation was carried out by three Portuguese researchers who are fluent in Portuguese and English. The questionnaire was then back translated into English by a native English-speaking researcher, unrelated to this study. No relevant inconsistencies were found between the back-translation and the original versions, indicating that the Portuguese version of the SABS had the same or very similar meaning as the original version. The final version of the questionnaire was then qualitatively tested in a sample of 20 female college students to assure its suitability, and slight phrasing changes were made. Analyses of the psychometric properties of the SABS with the current sample will be presented in the results section.

*Rape Myths Scale* (RMS; Martins et al., 2012). The RMS is a 30-item self-report measure that evaluates the acceptance of stereotypical and prejudicial beliefs regarding rape (e.g., “Most of the times, offenders are unknow to the victims”). Respondents are asked to express their agreement with each item, using a 5-point scale, ranging from 1 = strongly disagree and 5 = strongly agree. The original version of the scale revealed a high internal consistency (α = .91) and one factor solution (Martins et al., 2012). In the current study, internal consistency was .93.

*Youth Psychopathic Traits Inventory—Short Version* (YPI-S; Van Baardewijk et al., 2010; Portuguese version: Pechorro et al., 2015). The YPI-S is an 18-item self-report shorter version of the original scale (Andershed et al., 2002) designed to measure psychopathic traits, such as grandiosity-manipulative (e.g., “I am good at getting people to believe me when I make something up”), callous-unemotional (e.g., “I think that crying is a sign of weakness, even if no one sees you”), and impulsive-irresponsible (e.g., “I consider myself as a pretty impulsive person”). Each item is scored on a 4-point Likert scale ranging from “0 = Does not apply at all” to “3 = Applies very well.” The psychometric properties of the YPI-S have been examined among community and forensic samples (e.g., Pechorro et al., 2015, 2017), with internal consistency values ranging between 0.67 and 0.84. The scale has been used and investigated with adult samples (e.g., Colins & Andershed, 2016), also presenting good psychometrics. In the current study, the following alphas were found: .79 for the grandiosity/manipulative; .78 for callous-unemotional and impulsive-irresponsible dimensions.

*Personal Assessment of Intimacy in Relationships Scale* (PAIR; Schaefer & Olson, 1981; Portuguese version: Moreira et al., 2009). The PAIR is a 36-item self-report questionnaire aimed at assessing the perception of intimacy in relationships, namely personal validation (i.e., validation/acceptance by the partner; e.g., “I feel put-down in a serious conversation with my partner”), communication (i.e., expressing opinions, feelings, and desires within the relationship; e.g., “I can state my feelings without him/her getting defensive”), and openness to outward (i.e., sharing time with the peer group; e.g., “Having time together with our friends is an important part of our shared activities”). Schaefer and Olson (1981) provided both internal reliability and a factor structure for the PAIR. The Portuguese version of the scale presented good internal consistency values, ranging from 0.71 to 0.92 (Moreira et al., 2009). In the present sample, the Cronbach alphas were as follows: .91 for openness, .88 for personal validation, and .77 for communication.

### Data Analysis

Firstly, descriptive statistics (frequencies and percentages) were calculated for each item of the SABS. As the online survey required mandatory responses, no missing data was found. Afterwards, the total sample (*N* = 1,632) was randomly split into three subsamples, 30% (*n* = 490), 35% (*n* = 571), and 35% (*n* = 571) using the IBM-SPSS (version 26 for windows). The first 30% subsample was used for an EFA (calibration sample), the second 35% subsample for a CFA (first validation sample), and the third 35% subsample for an additional “twin” CFA (second validation sample), that is a CFA where the findings of the previous CFA were cross-checked in a subsample of equal power (Kyriazos, 2018). All subsamples presented adequate size as we guaranteed more 20 cases per item (Costello & Osborne, 2005; Schumacker & Lomax, 2015). We verified the KMO sampling adequacy values, the Barlett’s test of sphericity and high correlations between items (*r* > .9) to avoid multicollinearity issues (Field, 2013). An EFA based on tetrachoric correlation matrix was performed to discover the underlying factorial structure of the SABS items using FACTOR software version 10.10.02 (Lorenzo-Seva & Ferrando, 2006–2019). Before AFE, the number of latent factors were determined based on three methods: (1) Optimal implementation of parallel analysis (PA) with 2,000 random samples (Timmerman & Lorenzo-Seva, 2011); (2) Velicer’s (1976) minimum average partial test (MAP), and (3) Hull method (Lorenzo-Seva et al., 2011). Afterwards, an EFA using the Weighted Least Square Mean and Variance Adjusted (WLSMV) estimation method with a PROMIN oblique rotation (if necessary) was performed (Pires et al., 2019; Timmerman & Lorenzo-Seva, 2011). Only items with factor loadings higher than 0.3 were representative (Field, 2013).

We also assessed construct replicability (which can be defined as the proportion of the factor variance that can be accounted for by its indicators) using the H index, ranging from 0 to1, with acceptable values when *H* ≥ 0.70 (Hancock & Mueller, 2001).

The closeness to unidimensionality was also examined through the mean of item residual absolute loadings (MIREAL) and the explained common variance (ECV). MIREAL is a measure of departure from unidimensionality, with values lower than 0.30 indicating no substantial bias if a unidimensional solution is assumed (Ferrando & Lorenzo-Seva, 2018). Regarding ECV, values should be larger than 0.70 for a unidimensional solution (Rodriguez et al., 2016).

After finding the final optimal factor structure, a Confirmatory Factor Analysis (CFA 1) was performed using the second subsample. Considering that the observed variables were dichotomous, the weighted least square mean and variance adjusted estimator (WLSMV) with delta parameterization was used. A second “twin” (CFA 2) using the third subsample was performed to crosscheck the findings of CFA 1. To evaluate the overall fit of the factorial model in both subsamples, four indices were selected: (a) the Chi-Square (χ^2^) statistic; (b) the Comparative Fit Index (CFI; Bentler, 1990); (c) the Tucker-Lewis index (TLI; Bentler, 1990); and (d) the root mean square error of approximation (RMSEA; Steiger, 1990). The following criteria were used as cutoffs for good fit: a non-significant Chi-Square (χ^2^) statistic, CFI and TLI > 0.90 (with >0.95 being ideal), and RMSEA < 0.08 (with <0.05 being ideal; Brown, 2006). Considering the downfalls and misspecifications that can arise from revising a model based on Modification Indices (MIs; MacCallum, 1986; Silvia & MacCallum, 1988), MIs were not used to improve models fit to avoid increasing the risk of type I and type II errors (Ullman, 2006). CFAs were performed using structural equation modeling with the Mplus 8.3 software (Muthén & Muthén, 2017).

After establishing the model with better fit to the data, Kuder-Richardson 20 (KR-20) and McDonald’s omega (ωt) were computed as indices of internal consistency for the three subsamples and total sample. The minimum threshold of .60 was considered acceptable for both KR-20 and ωt (Bagozzi & Yi, 1988, 2012; DeVellis, 2017; Gliner et al., 2017; Hair et al.,2014). The average inter-item correlation (AIIC) was used to assess items homogeneity. The average item-rest correlation (AIRC) allowed us to examine the discriminating power of items. Pearson *r* values of .10, .30, and .50 were considered small, medium, and large in magnitude, respectively, as recommended by Cohen (1988).

Finally, both convergent and discriminant validity were examined for the total sample. Convergent validity was assessed at individual item and construct level based in three criteria: (a) the factor loadings should be statistically significant (*p* < .05); (b) average variance extracted (AVE) of the construct should be above the recommended cut-off .50; and (c) reliability coefficients > .60. AVE was manually computed following the guidelines by Fornell and Larcker (1981). Then, associations between the SABS, rape myths, and psychopathic traits scores were tested using Pearson’s correlations. In this sense, the hypothesis is that the correlations for testing convergent validity are statistically significant and moderate (Chin & Yao, 2014). Discriminant validity was assessed through the association between the SABS score and the perception of intimacy in relationships. The threshold of |*r*| ≥ .30 was used as the cutoff for evidence of convergence and |*r*| < .30 was used as the cutoff for evidence of divergence (Marczyk et al., 2005). In all statistical procedures, a significance level of .05 was set.

## Results

### Descriptive Statistics

In Table 1, responses (absolute frequencies and percentages) to the 12 items of SABS for the entire sample are reported. As shown, most respondents (more than 90%) neither reported engaging in coercive or abusive behaviors nor using physical force. It should be noted that only 0.4% of the respondents mentioned they have attempted to have sexual contact with man by threating him with a weapon (item 12), being the least frequent behavior.

**Table 1.** Descriptive Statistics for Responses to Items of the SABS (*N* = 1,632).

	The behavior has never occurred		The behavior has occurred at least once	
Items	*n*	%	*n*	%
1. How many times have you attempted to have sexual contact with a man by threatening to end your relationship?	1581	96.9	51	3.1
2. How many times have you attempted to have sexual contact with a man by saying things that you didn’t mean?	1403	86.0	229	14.0
3. How many times have you attempted to have sexual contact with a man by pressuring him with verbal arguments?	1485	95.5	147	9.0
4. How many times have you attempted to have sexual contact with a man by questioning his sexuality (suggesting that he may be impotent or gay)?	1558	95.4	74	4.5
5. How many times have you attempted to have sexual contact with a man by threatening to harm yourself?	1614	98.9	18	1.1
6. How many times have you attempted to have sexual contact with a man by using your position of power or authority (boss, teacher, babysitter, counselor, or supervisor)?	1600	98.0	32	2.0
7. How many times have you attempted to have sexual contact with a boy between 12 and 18 years of age who was five or more years younger than yourself?	1519	93.1	113	6.9
8. How many times have you attempted to have sexual contact with a man by getting him drunk or high?	1597	97.9	35	2.1
9. How many times have you attempted to have sexual contact with a man by taking advantage of a compromising position he was in (being where he did not belong or breaking some rule)?	1493	91.5	139	8.5
10. How many times have you attempted to have sexual contact with a man by threatening to use some degree of physical force (holding him down, hitting him, etc.)?	1591	97.5	41	2.5
11. How many times have you attempted to have sexual contact with a man by using some degree of physical force?	1573	96.4	59	3.6
12. How many times have you attempted to have sexual contact with man by threatening him with a weapon?	1626	99.6	6	0.4

### Exploratory Factor Analysis (EFA)

Following the guidelines by Field (2013), the calibration sample data (*n* = 490) was checked for items with zero or near-zero variance. As item 12 “*How many times have you attempted to have sexual contact with a man by threatening him with a weapon?*” showed a variance close to 0 (*S*² = 0.002), it was excluded from further analyses. Moreover, item 11 “*How many times have you attempted to have sexual contact with a man by using some degree of physical force?*” showed high multicollinearity with item 10 (*r* = .998) and was removed from the analysis. The PA (based on the 95% percentile) in conjunction with MAP test and the Hull method suggested one-factor structure. After, the AFE was performed as the KMO value of 0.94 indicated good adequacy of the tetrachoric correlation matrix. The significance of Bartlett’s sphericity test [χ^2^ (45) = 5,581.90; *p* < .001] revealed that the correlations between items were adequate to conduct an EFA. As seen in table 2, results pointed out to a stable one-factor structure with factor loadings ranging from 0.40 to 0.79.

**Table 2.** One-Factor Structure of the Portuguese Version of SABS (*n* = 490).

Items	Factor (sexual aggression)	*h* ^2^
Item 1—How many times have you attempted to have sexual contact with a man by threatening to end your relationship?	0.79	0.62
Item 5—How many times have you attempted to have sexual contact with a man by threatening to harm yourself?	0.76	0.57
Item 9—How many times have you attempted to have sexual contact with a man by taking advantage of a compromising position he was in (being where he did not belong or breaking some rule)?	0.71	0.50
Item 4—How many times have you attempted to have sexual contact with a man by questioning his sexuality (suggesting that he may be impotent or gay)?	0.70	0.50
Item 8—How many times have you attempted to have sexual contact with a man by getting him drunk or high?	0.69	0.48
Item 2—How many times have you attempted to have sexual contact with a man by saying things that you didn’t mean?	.68	0.46
Item 6—How many times have you attempted to have sexual contact with a man by using your position of power or authority (boss, teacher, babysitter, counselor, or supervisor)?	0.66	0.44
Item 10—How many times have you attempted to have sexual contact with a man by threatening to use some degree of physical force (holding him down, hitting him, etc.)?	0.66	0.44
Item 3—How many times have you attempted to have sexual contact with a man by pressuring him with verbal arguments?	0.56	0.31
Item 7—How many times have you attempted to have sexual contact with a boy between 12 and 18 years of age who was five or more years younger than yourself?	0.40	0.16

*Note*. Extraction method: WLSMV; *h*^2^: communality; Item loadings are sorted in decreasing order.

The latent factor found was labeled “Sexual Aggression,” consisting of 10 items and explaining more than 50% of the variance, as recommend by Streiner (1994).

Construct replicability was good (*H* = 0.90). The MIREAL and ECV values were 0.26 and 0.83, respectively, supporting a unidimensional solution (García-Campayoet al., 2018; Rodriguez et al., 2016).

### Confirmatory Factor Analyses

For the first validation sample (*n* = 571), the one-factor model presented a good fit to the data, with a χ^2^ (35) =  44.64; *p* = .127, TLI = 0.95 and CFI = 0.94, and a RMSEA of .02, as seen in Table 3. Similar results were found for second validation sample (twin CFA) with χ^2^ (35) = 41.95; *p* = .195, TLI = 0.98, CFI = 0.97, and a RMSEA of 0.02, demonstrating that the one-factor solution is replicable (stable) and generalizable (DeVellis, 2017).

**Table 3.** Fit Indices for Confirmatory Factor Analyses.

	χ^2^ (*df*)	*p*	TLI	CFI	RMSEA [90% CI]
CFA 1 (*n* = 571)	44.64 (35)	.127	0.95	0.94	0.02 [0.00–0.04]
CFA 2 (*n* = 571)	41.95 (35)	0.195	0.98	0.97	0.02 [0.00–0.04]

*Note*. χ^2^ = chi-square test based using the WLSMV estimator; *df* = degrees of freedom; *p* = *p*-value; TLI = Tucker-Lewis Index; CFI = comparative fit index; RMSEA = root mean square error of approximation.

Table 3 summarizes the fit indices for the two validation samples (CFA 1 and CFA 2) using the WLSMV estimation method for the tetrachoric correlation matrix of the SABS items.

**Figure 1.**
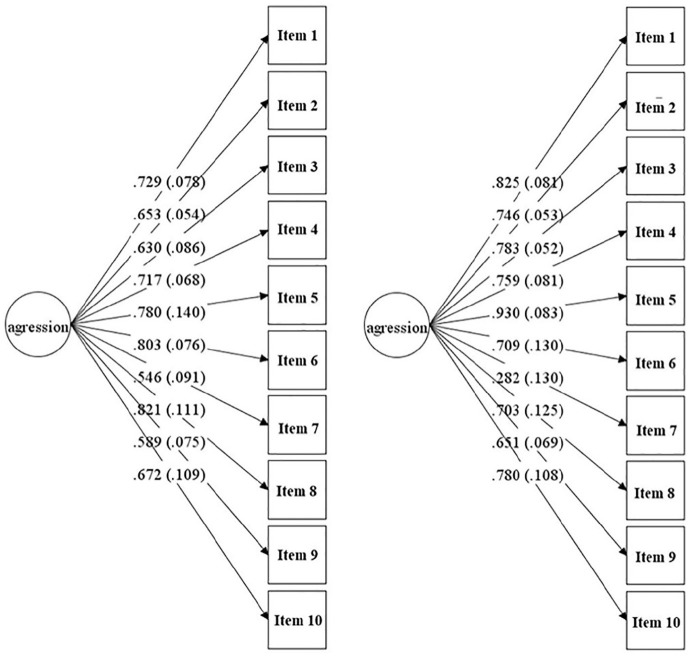
The single factorial structure of the Portuguese version of SABS for CFA 1 (left) and CFA 2 “twin CFA” (right). Values represent standardized regression weights and standard error within brackets.

As presented in Figure 1, the standardized factor loadings for both CFAs satisfied the requirement (λ > .50), except for item 7 in CFA 2. All standardized factor loadings were statistically significant with *p* < .001.

### Reliability

Table 4 presents the reliability results for all the samples. As shown, KR-20 and McDonald’s omega values were acceptable (Bagozzi & Yi, 2012; Gliner et al., 2017; Hair et al., 2014). All average inter-item correlations were within the adequate range (Clark & Watson, 1995), suggesting that the SABS is composed of reasonably homogenous items (Piedmont, 2014). With regard to average item-rest correlations (AIRC) were all positive and above .30 across all samples. All items (except for item 7 in the EFA sample) were not less than the rule of thumb minimum value of .20 (Kline, 1986), It is worth to note that the percentage of explained variance in AFE dropped after item 7 was excluded. Furthermore, the deletion of the item with the lowest item–whole correlation did not result in a substantial increase in the α coefficient. Therefore, the item 7 was included in the scale.

**Table 4.** Reliability, Homogeneity, and Discriminative Power of Items.

	ω*t*	KR-20	AIIC	AIRC [range]
First subsample (EFA; *n* = 490)	.62	0.62	.17	.31 [.19, .40]
First subsample (CFA 1; *n* = 571)	.64	0.65	.18	.33 [.26, .38]
Third subsample (CFA 2; *n* = 571)	.65	0.63	.17	.34 [.21, .43]
Total sample (*N* = 1,632)	.64	0.64	.17	.33 [.22, .37]

*Note*. ω*t* = McDonald’s omega; KR-20 = Kuder-Richardson 20; AIIC = Average Inter-Item Correlation; AIRC = Average Item-Rest Correlation.

### Convergent and Discriminant Validity

Convergent was examined using first criterion for convergent validity, that is, all λ > .50. As seen in figure 1., all factor loadings were higher than .50., except for item 7 in CFA 2, yet, still significant (*p* < .05). Regarding the second criterion for convergent validity, AVE was .49 and .54 for CFA 1 and CFA 2, respectively. Despite AVE was less than 0.5 for CFA 1, the McDonald’s omega was higher than 0.6, so the convergent validity of the construct is still acceptable (e.g., Fornell & Larcker, 1981; Tomás & Rosa, 2021). Concerning the third criterion, all reliability coefficients were above .60 across the three subsamples and for total sample (*N* = 1,632), supporting the convergent validity of the Portuguese version of SABS.

Convergent validity was further supported by the positive, statistically significant correlations between the SABS, rape myths, psychopathic traits, and personal validation (negative dimension measured by the PAIR) scores, ranging from weak to moderate associations (see Table 5). More precisely, the SABS score was significantly correlated to rape myths. Positive and significant associations were found between SABS and psychopathic traits, and the magnitude of correlations was medium. Finally, aggression was positively associated with personal validation, and the magnitude of correlations was small.

**Table 5.** Correlation Values Between the SABS, RMS, YPI, and PAIR Dimensions.

	Sexual aggression
Convergent validity	
RMS—rape myths	.23***
YPI—Grandiosity-manipulative	.35***
YPI—Callous-unemotional	.17**
YPI—Impulsive-irresponsible	.32***
Discriminant validity	
PAIR—Personal validation	−.18**
PAIR—Communication	−.27**
PAIR—Openness	−.11*

*Note*. RMS = Rape Myths Scale; YPI = Youth Psychopathic Traits Inventory; PAIR = Personal Assessment of Intimate Relationships Scale.

* *p* < .05. **p < .01. ***p < .001.

In terms of discriminant validity, the correlation coefficients between SABS and PAIR scores were statistically significant but weak, meeting the conventional criterion (|*r*| < .30) to support discriminant validity (Marczyk et al., 2005).

## Discussion

The main goal of this study was to assess the psychometric properties of the SABS using a large sample of female college students. Particularly, the internal structure of the scale was investigated, along with analyses of the internal consistency, and validity evidence in relation to external variables, namely rape myths, psychopathic traits, and perception of intimacy. We intended to further contribute to the validation of the SABS by conducting both an exploratory and confirmatory analyses, thus ascertaining their applicability to female college students.

Concerning the internal structure of the SABS, we found evidence for a single factor solution consisting of 10 items. Considering the strong overlap among the sexually aggressive strategies in samples of community women (e.g., Anderson, 1996), this one-factor solution (i.e., sexual aggression) may be more appropriate when using with female college students. Items 11 (“*How many times have you attempted to have sexual contact with a man by using some degree of physical force?*”) and 12 (“*How many times have you attempted to have sexual contact with a man by threatening him with a weapon?*”) showed multicollinearity problems and variance close to 0, respectively, and were excluded. It should be noted that these two items are related to hands-on strategies (i.e., physical force), which are the tactics less used by sexually aggressive women (Carvalho & Brazão, 2021). Although SV perpetrated by women are still understudied, it is unanimous that sexually aggressive women resort mainly to hands-off strategies, that is, sexual coercion and sexual abuse (Turchik, 2012; Carvalho & Nobre, 2016; Carvalho et al., 2018). Therefore, this 10-item version of SABS may be a more idiosyncratic, reliable, and robust tool to assess sexual aggression as committed by community women.

Convergent validity was supported by the positive and moderate associations between the SABS scores and rape myths and psychopathic traits, which is in line with previous findings (Beech et al., 2009; Bouffard et al., 2016; Gannon et al., 2010). Nevertheless, the association between SABS scores and rape myths was small, thus not establishing a substance level of convergence (Gregory, 2007). This result suggests that rape myths do not play a major role in women’s SV. In fact, research has shown that rape myths are accepted by non-violent individuals, being highly prevalent in the general population, but also among counselors, medical trainees, rape crisis workers, and professionals in the justice system (Anderson & Quinn, 2009; Struckman-Johnson & Struckman-Johnson, 1993; Donnelly & Kenyon, 1996; Kassing & Prieto, 2003; Turchik & Edwards, 2012; Walfield, 2021). Another concurrent explanation for this result may have to do with the self-report measure used to assess rape myths. Although we used an instrument gendered-neutral, most items do not assess specific myths about SV against men (e.g., “real” men can defend themselves against rape). It could be expected that these specific myths and women’s SV would be more strongly associated. To clarify this issue, future research should test the association between women’s SV and male rape myths, as measured, for instance, by the Male Rape Myth Scale (Melanson, 1998).

Discriminant validity was found between the SABS and PAIR scores. Specifically, sexual aggression was negatively associated with personal validation. This result suggest that sexual aggression may be used by women who do not feel validated or accepted by the partner, which reinforces the idea that women’s SV may be used to regulate emotional states or fulfil intimacy needs (Gannon et al., 2008; Nathan & Ward, 2002). Besides the weak correlation values we found, the results of divergent validity showed negative associations between sexual aggression and communication and openness, suggesting that intimacy difficulties, namely at the communication level (expressing opinions, feelings, and desires within the relationship) may underlie women’s sexual violence against intimate partners, as observed in criminal samples of female sex offenders (e.g., Gannon et al., 2008; Nathan & Ward, 2002).

This research is not without limitations, namely the fact that only included female college students. Therefore, the present findings relate to a particular population living in a specific social context. No generalizations should be made to other samples (e.g., women from other age-groups/contexts, female sex offenders). It is important to add that SABS items assessed attempted, rather than consummated sexual aggression. So, there is no information on the outcomes of using such tactics. In addition, there is no data on the men’s level of consent. Also, future studies conducted with SABS should include a sample of men as initiators of attempted sexual aggression (by adapting the SABS items), allowing testing measurement invariance across gender, as well as comparisons concerning the use of sexually aggressive behaviors toward an unwilling partner. In addition, developing and assessing the psychometric properties of instruments aimed to capture SV dynamics in sexual minorities seems of the utmost importance.

Overall, this study contributed to validating the SABS for use with female college students. Although research about women’s SV is scarce, the available findings indicate that community women do use sexually violent behaviors, particularly hands-off strategies (e.g., Carvalho & Nobre, 2016; Carvalho et al., 2018). Therefore, is paramount to develop credible and valid instruments that may accurately address these sexually aggressive behaviors as committed by community women. The current research supports the use of this instrument with female college students and provides the researcher/clinician an idiosyncratic and reliable tool for assessing women’s sexually aggressive behaviors toward men. Moreover, considering the significant prevalence of SV perpetrated by female college students (e.g., Bouffard et al., 2016; Marshall et al., 2021; Miller et al., 2017), developing and delivering SV prevention programs in college campuses seems of the utmost importance, and SABS could be used as a reliable outcome measure aiming to assess the program’s efficacy in reducing SV perpetration.
